# Evidence of gut enteropathy and factors associated with undernutrition among slum-dwelling adults in Bangladesh

**DOI:** 10.1093/ajcn/nqz327

**Published:** 2020-01-07

**Authors:** Shah Mohammad Fahim, Subhasish Das, Md Amran Gazi, Md Ashraful Alam, Mustafa Mahfuz, Tahmeed Ahmed

**Affiliations:** 1 Nutrition and Clinical Services Division, International Centre for Diarrhoeal Disease Research, Bangladesh (icddr,b), Dhaka, Bangladesh; 2 Faculty of Medicine and Life Sciences, University of Tampere, Tanpere, Finland; 3 Department of Global Health, University of Washington, Seattle, WA, USA; 4 James P Grant School of Public Health, BRAC University, Dhaka, Bangladesh

**Keywords:** undernutrition, BMI, micronutrient deficiencies, dietary diversity, anemia, α-1 antitrypsin, gut enteropathy, SRQ-20, adults, Bangladesh

## Abstract

**Background:**

Adult undernutrition (BMI <18.5 kg/m^2^) is responsible for immune deficits, increased risk of disease burden, and higher rates of mortality. The prevalence of adult undernutrition in Bangladesh is substantial, but there have been few studies on the etiology of this condition for the inhabitants of urban slums.

**Objective:**

The aim of this study was to identify the factors associated with undernutrition among slum-dwelling adults in Bangladesh.

**Methods:**

A case-control study was conducted in the Bauniabadh area of Dhaka, Bangladesh. 270 adult participants (135 cases with a BMI <18.5 and 135 controls with a BMI between 18.5 and 24.9) aged 18–45 y were enrolled between October 2018 and January 2019. Sociodemographic variables, dietary diversity, micronutrient deficiencies, psychological symptoms, infection, and biomarkers of gut health were assessed to identify the factors associated with undernutrition using multivariable logistic regression analysis.

**Results:**

A higher number of siblings [adjusted odds ratio (aOR): 1.39; 95% CI: 1.11, 1.77], increased self-reporting questionnaire-20 score (an instrument to screen mental health disorders and detect psychological symptoms) (aOR: 1.12; 95% CI: 1.04, 1.23), elevated fecal concentration of α-1 antitrypsin (aOR: 4.82; 95% CI: 1.01, 25.29), and anemia (aOR: 3.63; 95% CI: 1.62, 8.58) were positively associated with undernutrition in adults. Age (aOR: 0.90; 95% CI: 0.84, 0.96), dietary diversity score (aOR: 0.75; 95% CI: 0.56, 0.99), C-reactive protein (aOR: 0.82; 95% CI: 0.73, 0.92), *Helicobacter pylori* infection (aOR: 0.11; 95% CI: 0.05, 0.23), and always washing hands before eating or preparing foods (aOR: 0.33; 95% CI: 0.12, 0.87) were associated with reduced odds of undernutrition among the study population.

**Conclusions:**

Our results indicate that undernutrition in slum-dwelling adults in Bangladesh is associated with numerous physiological and sociodemographic factors, including evidence of gastrointestinal inflammation and altered intestinal permeability.

## Introduction

Undernutrition in adults, which is characterized by low BMI (<18.5 kg/m^2^), has been associated with reduced productivity, lower IQ, and impaired economic development (1–4). The WHO estimated that globally there were 462 million underweight adults in 2014 (5), but there are currently no global targets to address this pressing problem. Having a BMI <18.5 weakens the immune system, increases risk of infectious diseases, alters body composition, accelerates growth impairment, and leads to higher rates of disability, morbidity, and mortality (6–10). Undernutrition during adulthood signifies the presence of chronic energy deficiency (CED) (11). Studies confirm that CED, a state of low energy store and lean body mass, contributes to decreased work capacity among adults, which results in reduced national productivity and halts economic growth (1, 12). For men, undernutrition is indicative of reduced economic ability, poverty, food insecurity, and lack of access to adequate health care services (13–15). A study conducted in Bangladesh suggested an increase in the morbidity rates among adult males who are underweight (16).

Low BMI among women is an important determinant of poor reproductive health (17). Maternal undernutrition results in inadequate fetal nutrition followed by adverse obstetric and neonatal outcomes (18, 19). Undernourished mothers are more likely to have adverse pregnancy events, including obstructed labor and postpartum hemorrhage (7, 20). Underweight girls have greater risk of delivering small and low birth weight babies (21). The challenge is particularly acute in South Asia, where it is estimated that almost 40% of adolescent girls are underweight and this number is falling by only about 1% per year (22). Hence, approaches that target adult undernutrition, in parallel with those aimed at infants and children, are essential to help break the intergenerational cycle of malnutrition and its devastating consequences.

Bangladesh has experienced a reduction in the prevalence of undernutrition over the past 2 decades (23). However, a significant proportion of the adult population in the country is underweight (24). According to the 2014 Bangladesh Demographic and Health Survey (BDHS), 19% of ever-married Bangladeshi women aged 15–49 y were underweight (23), while data are lacking for men and unmarried women. Undernutrition is a complex phenomenon influenced by several factors, including dietary diversity, micronutrient status, psychological symptoms, infection, and altered gut health. While risk factors for childhood undernutrition in the country have been described (25, 26), factors contributing to adult undernutrition are less well understood, especially among those living in urban slums. Previous studies have used secondary data and only highlighted a small number of sociodemographic correlates (24, 27–29). Therefore, in this study, we aimed to identify the factors associated with undernutrition among slum-dwelling adults living in Dhaka, Bangladesh.

## Methods

### Study design and setting

We performed a case-control study conducted among the residents of the Bauniabadh area of Mirpur, a resource-poor urban settlement in Dhaka, Bangladesh. The detailed sociodemographic information of the study location has been published elsewhere (30, 31). In this study, cases were defined as participants having a BMI <18.5, and adults with a normal BMI (18.5–24.9) were used as controls. Screening for adult participants aged 18 to 45 y with a BMI between 16.0 and 24.9 was done through household visits in the community. Subjects were excluded if another family member was enrolled in the same study, if they were suffering from severe disease requiring hospitalization, or if they presented with a congenital anomaly or chromosomal abnormality, were pregnant or lactating women, or had a chronic illness. The eligible participants were invited to participate in the study and offered incentives to compensate for their wage loss owing to visiting the study office for providing biological samples and facilitating the data collection. After detailed explanation of the study procedure by trained field staff, those who were willing to participate were enrolled in the study. A total of 270 adult participants (135 cases and 135 controls) aged 18 to 45 y were enrolled from October 2018 to January 2019.

### Ethics

The study protocol was reviewed and approved by the Institutional Review Boards at the International Centre for Diarrhoeal Diseases and Research, Bangladesh (icddr,b). All participants provided informed written consent.

### Data collection

After enrollment, anthropometry data were collected by trained field staff from all the participants. Socioeconomic status, an FFQ, and a self-reporting questionnaire-20 (SRQ-20) were collected from all the participants at enrollment. SRQ-20 is a validated instrument to screen mental health disorders and detect psychological symptoms in adults (32, 33). Whole venous blood (2 mL) was collected aseptically by venipuncture for assays of micronutrient status, levels of C-reactive protein (CRP), and α-1-acid glycoprotein (AGP). Stool samples were collected during enrollment from the participants for *Helicobacter pylori* stool antigen testing and for quantification of biomarkers of intestinal inflammation and gut barrier integrity [myeloperoxidase (MPO), neopterin (NEO), and α-1 antitrypsin (AAT)].

### Laboratory analyses

Laboratory analyses were carried out at icddr,b. Blood samples were collected in EDTA tubes and centrifuged (3000 × *g*; 10 min; 20°C) within 2 h of collection to separate the plasma. Aliquots were stored in −80°C freezers until analysis. Plasma CRP (Immundiagnostik), AGP (Alpco), and ferritin (ORGENTEC Diagnostika GmbH) were quantified using commercially available ELISA kits following the manufacturers’ instructions. Atomic absorption spectrometry was used to measure plasma zinc concentrations. Stool biomarkers were quantified using commercially available kits [MPO (Alpco), NEO (GenWay Biotech), and AAT (Biovendor)]. *H. pylori* antigen was quantified in stool samples using Amplified IDEIA™ Hp StAR™ (OXOID Limited), where an absorbance value ≥0.15 was considered positive for *H. pylori* infection.

### Variables used in this analysis

The presence or absence of undernutrition in adults (BMI <18.5) was the outcome variable used in our analysis. Sex, mode of delivery at birth, treatment and source of drinking water, access to improved sanitation, hand washing practices before cooking or taking meals, hand washing practices after using the toilet, use of toilet paper, separate space for kitchen, animal exposure in households, employment status, academic qualifications, household crowding index, anemia, iron deficiency, zinc deficiency, and *H. pylori* infection were the categorical variables. Anemia was defined by hemoglobin <12 g/dL for female and <13.5 g/dL for male study participants. Ferritin <12 ng/mL and zinc <0.7 mg/L were considered as iron deficiency and zinc deficiency, respectively. Improved water source and improved sanitation were defined per the criteria of the WHO (34). The household crowding index was categorized as low, medium, or high as previously described (35). Age, number of siblings, working hours, screen viewing hours, monthly family income, money spent on food in a month, dietary diversity score (DDS), and SRQ-20 score were the continuous variables included in this analysis. The DDS was calculated according to the guidelines for measuring household and individual dietary diversity prepared by the Food and Agriculture Organization of the United Nations (36). Since the DDS for women was validated in Bangladesh (37), we considered this score to calculate the DDS of our study participants. The SRQ-20 is a 20-question tool for assessing the depressive symptoms among adults (38, 39). Markers of systemic inflammation (CRP and AGP), intestinal inflammation (MPO and NEO), and intestinal permeability (AAT) were also included as covariates in this analysis.

### Statistical analysis

Demographic and socioeconomic characteristics were expressed as the mean ± SD for normally distributed quantitative variables or as the median and IQR for asymmetric quantitative variables. Frequency with a proportion estimate was used for categorical variables. To assess the statistical significance of differences between the groups, a *t* test was performed for mean values, and the Mann-Whitney U test was used for median values. Differences in proportions between the groups were tested with Pearson's chi-square test. Spearman's correlation coefficients were calculated to determine the correlation between BMI and different biomarkers. A multivariable logistic regression model was used to identify the factors associated with undernutrition in adults. Variables with *P* values <0.20 in the univariate logistic regression analysis were included in the multivariable model. The association was expressed as the adjusted odds ratio (aOR) and 95% CI. A *P* value of <0.05 was considered statistically significant for the multivariable logistic regression model.

Regression diagnostics were used to evaluate the assumptions and assess the validity of the logistic regression model. We performed the Hosmer–Lemeshow goodness of fit test for the multivariable logistic regression model applied in this analysis. An insignificant *P* (0.53) indicated that the model had good fit. Multicollinearity was excluded, with all the variance inflation factor (VIF) values being <2. A receiver operating characteristic (ROC) curve was produced to measure the accuracy of the model. We tested for assumption of linearity to assess whether log odds of the outcome was linearly associated with the explanatory variables and found a linear relationship between the exposures and outcome. An insignificant hatsq (*P* = 0.56) in linktest demonstrated that the link function was correctly specified and there was no specification error in our logistic regression model. The McFadden's pseudo *R*^2^ value of the model was 0.46. All the analyses were performed using R version 3.5.3 software (https://www.r-project.org, Foundation for Statistical Computing).

## Results

**FIGURE 1:**
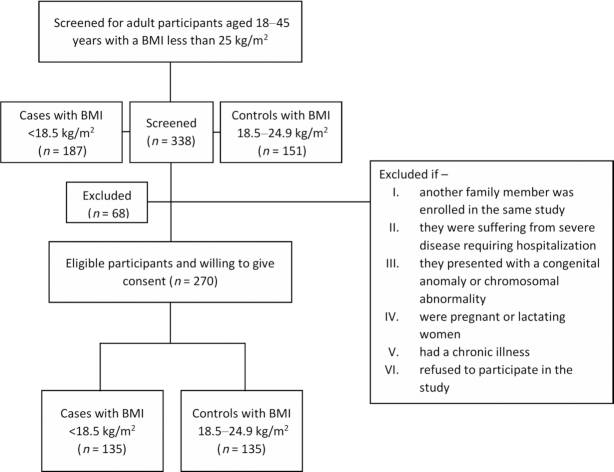
Flowchart showing the inclusion of cases and controls in this study.

Overall, 338 adults aged 18–45 y with BMI <25.0 were screened for this study, of whom 187 were undernourished and 151 were healthy. Of these individuals, 31 did not meet the eligibility criteria and 37 refused to be enrolled in the study. Among the 31 adults who were screened out according to the exclusion criteria, 29% were pregnant or lactating mothers, 19% were from the same households, 19% migrated from the study area, 13% were suffering from chronic illnesses, 10% had chromosomal abnormalities or congenital anomalies, and 10% were drug addicted. The reasons for refusals were as follows: refused to provide biological samples (16%), students unable to visit study office due to class attendance (11%), and inability to manage time from the employer to visit the study office (62%). Only 4 adults refused to participate without providing any specific reasons. The enrollment scheme is shown in **Figure 1**. A total of 270 adults were included in this analysis. Among these participants, 135 were undernourished adults and 135 were adults with normal BMI. **Table 1** provides the demographic and socioeconomic characteristics of the participants enrolled in this study. The mean ± SD age of the study participants was 24.2 ± 6.6 y, and 75.9% of the enrolled participants were women. The mean ± SD BMIs of the undernourished adults and healthy controls were 17.3 ± 0.7 and 21.8 ± 1.8, respectively; 87% of the participants had received formal education. The employment rate of the healthy controls was higher than that of the undernourished adults. Overall, 35.2% of participants were employed, with 7.4 ± 3.8 daily working hours. Screen viewing hours (mean ± SD) were greater in undernourished adults (2.5 ± 1.6 h) than in their healthy counterparts (2.1 ± 1.5 h). All of the participants had access to an improved source of water and 56.3% reported treating water regularly to make it safe for consumption. The monthly family income of the undernourished adults was lower than the corresponding control group. Based on SRQ-20 scores, depressive symptoms were more prevalent in undernourished adults than controls.

**TABLE 1:** Descriptive characteristics of the slum-dwelling adult participants^1^

Variable	Healthy adults (*n* = 135)	Undernourished adults (*n* = 135)	Overall (*n* = 270)
Age, y	25.1 ± 6.6	23.4 ± 6.6	24.2 ± 6.6
Female sex	95 (70.4)	110 (81.5)	205 (75.9)
BMI, kg/m^2^	21.8 ± 1.8	17.3 ± 0.7	19.6 ± 2.6
Education level			
No education	17 (12.6)	17 (12.6)	34 (12.6)
Primary	46 (34.1)	49 (36.3)	95 (35.2)
Secondary	51 (37.8)	47 (34.8)	98 (36.3)
Higher secondary and above	21 (15.5)	22 (16.3)	43 (15.9)
Mode of delivery			
Normal delivery	133 (98.5)	134 (99.3)	267 (98.9)
Caesarean section	2 (1.5)	1 (0.7)	3 (1.1)
Employment	55 (40.7)	40 (29.6)	95 (35.2)
Working hours	7.2 ± 4.1	7.6 ± 3.3	7.4 ± 3.8
Screen hours	2.1 ± 1.5	2.5 ± 1.6	2.3 ± 1.6
Smoker	18 (13.3)	6 (4.4)	24 (8.9)
Substance abuser	9 (6.7)	9 (6.7)	18 (6.7)
Improved water sources	135 (100)	135 (100)	270 (100)
Water treatment	72 (53.3)	80 (59.3)	152 (56.3)
Improved sanitation	28 (20.7)	29 (21.5)	57 (21.1)
Use of toilet paper	44 (32.6)	25 (18.5)	69 (25.6)
Always wash hands before meal	32 (27.8)	20 (15.2)	52 (21.1)
Always wash hands after toilet	95 (70.4)	106 (78.5)	201 (74.4)
Monthly family income, US$	248.3 ± 148.3	198.1 ± 116.2	223.2 ± 135.3
Money spent on food in a month, US$	109.6 ± 54.3	93.0 ± 43.7	101.3 ± 49.9
Number of siblings	3.1 ± 1.6	3.8 ± 1.8	3.5 ± 1.7
Household crowding index			
Low (0–1 people)	2 (1.5)	2 (1.5)	4 (1.5)
Medium (2–4 people)	116 (85.9)	109 (80.7)	225 (83.3)
High (>4 people)	17 (12.6)	24 (17.8)	41 (15.2)
Separate space for kitchen	114 (84.4)	115 (85.2)	229 (84.8)
Household animal exposure	9 (6.7)	6 (4.4)	15 (5.6)
DDS	4.4 ± 1.2	4.0 ± 1.3	4.2 ± 1.3
SRQ-20 score	5.0 ± 5.0	6.6 ± 4.1	5.8 ± 4.6
Hemoglobin, g/dL	13.2 ± 1.7	12.4 ± 1.7	12.8 ± 1.8
Ferritin, ng/mL	81.5 ± 71.0	61.7 ± 59.3	71.6 ± 66.1
Zinc, mg/L	0.76 ± 0.1	0.73 ± 0.1	0.74 ± 0.1

1 Values are *n* (%) or means ± SDs. DDS, dietary diversity score; SRQ-20, self-reporting questionnaire-20.

### Prevalence of micronutrient deficiencies and *H. pylori* infection

**FIGURE 2:**
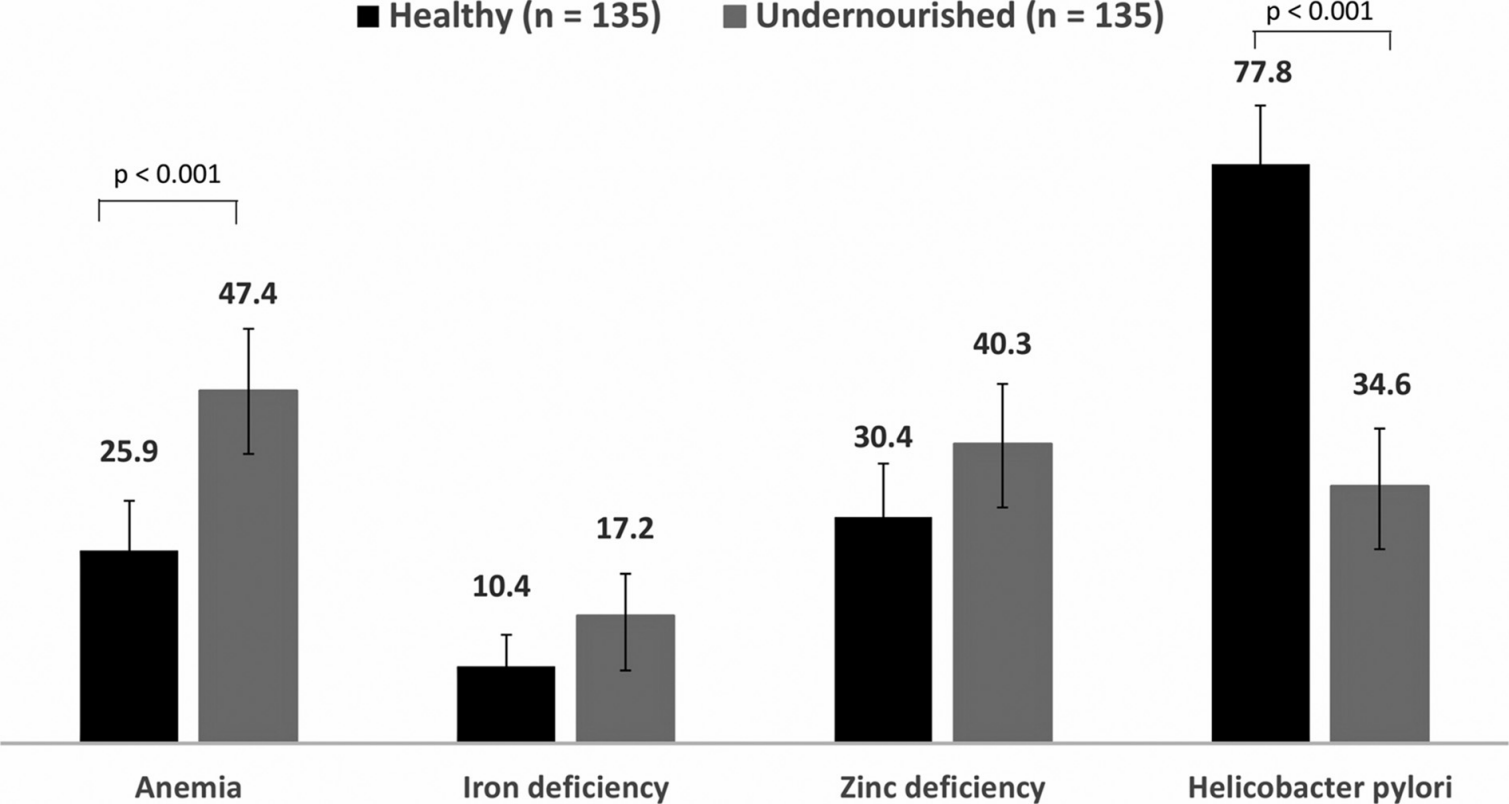
Prevalence of micronutrient deficiencies and *Helicobacter pylori* infection in slum-dwelling adults. Differences between the groups were tested with Pearson chi-square test. The prevalence of anemia, iron deficiency, and zinc deficiency was higher among undernourished adults, but the prevalence of *H. pylori* infection was higher in healthy adults. However, the differences in anemia and *H. pylori* showed statistical significance, *P* < 0.001.

The prevalence of different micronutrient deficiencies and *H. pylori* infection is presented in **Figure 2**. The prevalence of anemia was higher in the undernourished adults than the healthy controls, and the difference was statistically significant (*P*  < 0.001). Similarly, the rates of iron and zinc deficiencies were higher in the undernourished adults, although these differences were not statistically different. Conversely, the prevalence of *H. pylori* infection was significantly lower in the undernourished adults than the healthy controls (*P*  < 0.001).

### Levels of plasma and fecal biomarkers of inflammation and gut enteropathy

**FIGURE 3:**
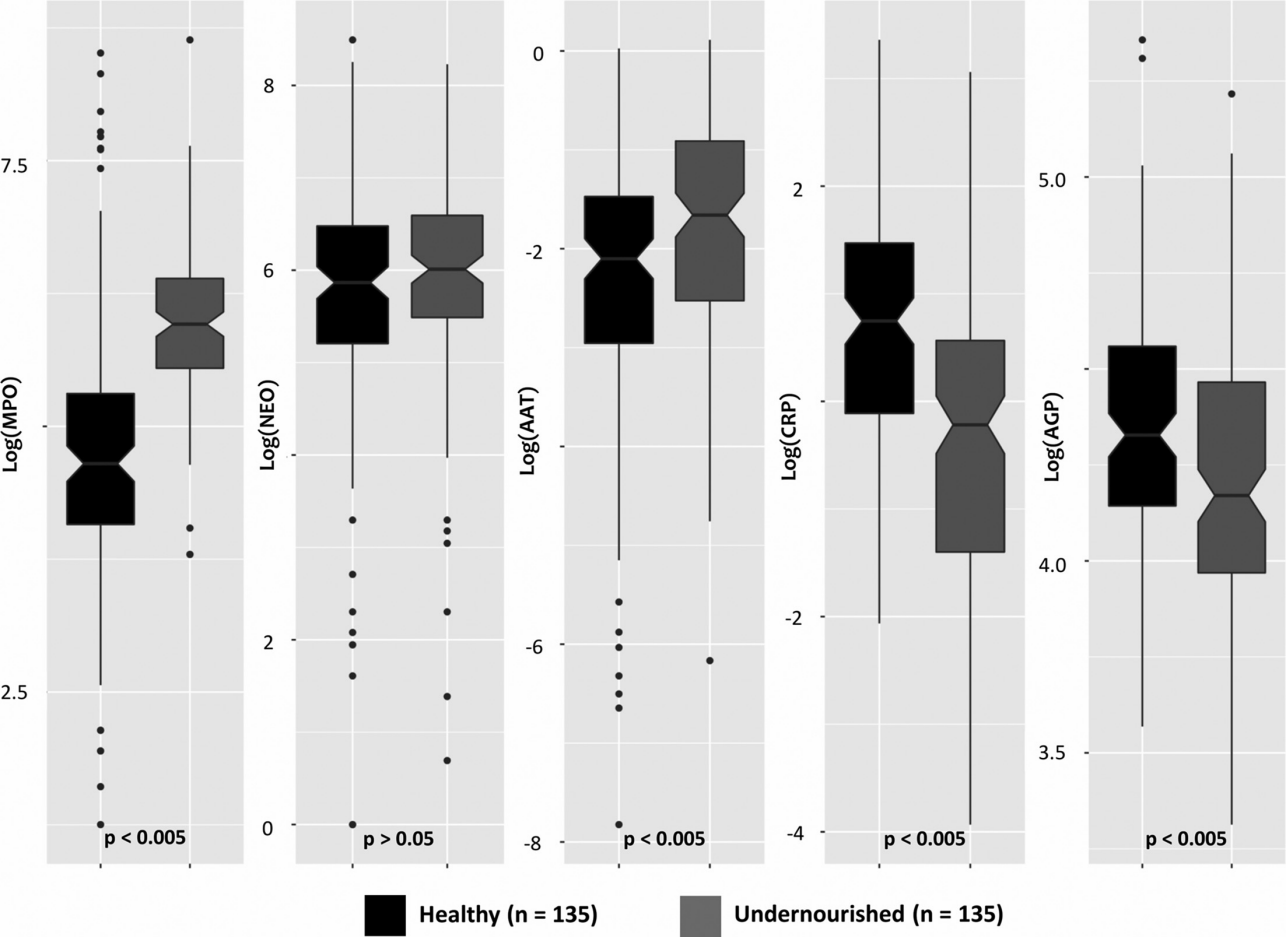
Distribution of biomarkers among the adult participants living in slums. Differences between the groups were tested with a Mann-Whitney test. Plasma concentrations of CRP and AGP were higher in healthy adults, *P* < 0.005. The concentrations of MPO, NEO, and AAT were all higher in the feces of undernourished adults, with the differences for MPO and AAT achieving statistical significance, *P*  < 0.005. AAT, α-1 antitrypsin; AGP, α-1-acid glycoprotein; CRP, C-reactive protein; MPO, myeloperoxidase; NEO, neopterin.

**Figure 3** illustrates the levels of plasma and fecal biomarkers measured in the 2 groups. The plasma concentrations of CRP and AGP were higher in healthy adults (*P*  < 0.005). Fecal biomarkers were present at higher concentrations than have been reported in studies in nontropical countries [<2000 ng/mL, <70 nmol/L, and <0.27 mg/g, respectively for MPO, NEO, and AAT (40)]. Overall, 3.4%, 90.7%, and 31.0% of the stool samples had higher concentrations of MPO, NEO, and AAT, respectively, than the reported values for nontropical countries (**Supplemental Figure 1**). Moreover, the concentrations of MPO, NEO, and AAT were all higher in the feces of undernourished adults (Figure 3), with the differences for MPO and AAT achieving statistical significance (*P*  < 0.005). **Supplemental Figure 2** displays multipanel scatter plots showing the correlations of plasma and fecal biomarkers with BMI of the adult participants in both the healthy and undernourished groups. Adult BMI exhibited significantly positive correlations with hemoglobin (*P* = 0.003), zinc (*P* = 0.048), CRP (*P* < 0.001), and AGP (*P* = 0.006) values measured in plasma. Negative correlations were observed between BMI and fecal biomarkers of gut enteropathy and were statistically significant for MPO (*P* < 0.001) and AAT (*P* = 0.005).

### Factors associated with undernutrition in adults

Multivariable logistic regression analysis (**Table 2**) showed that undernourished participants were on average younger than the normal-BMI controls (aOR: 0.90; 95% CI: 0.84, 0.96; *P* = 0.001). Likewise, undernourished adults had lower DDS (aOR: 0.75; 95% CI: 0.56, 0.99; *P* = 0.04) and CRP (aOR: 0.82; 95% CI: 0.73, 0.92; *P* = 0.001) values than the controls. Undernourished adults were more likely to have a greater number of siblings (aOR: 1.39; 95% CI: 1.11, 1.77; *P* = 0.005) and higher SRQ-20 scores (aOR: 1.12; 95% CI: 1.04, 1.23; *P* = 0.006). Undernutrition in adults was associated with greater likelihood of having elevated concentrations of fecal AAT (aOR: 4.82; 95% CI: 1.01, 25.29; *P* = 0.049). Compared with the normal-BMI adults, the aOR for anemia was 3.63 (95% CI: 1.62, 8.58; *P* = 0.002) among the undernourished adults. The odds of being undernourished were lower among the *H. pylori*–infected participants (aOR: 0.11; 95% CI: 0.05, 0.23; *P* < 0.001) and among those who always washed their hands before eating or preparing foods (aOR: 0.33 95% CI: 0.12, 0.87; *P* = 0.03). In contrast, the odds of undernutrition were higher among the participants who always wash their hands after using the toilet (aOR: 3.73; 95% CI: 1.48, 9.92; *P* = 0.006). The ROC curve illustrated that the overall predictive power of the estimated logistic regression model was 89.2% (**Supplemental Figure 3**).

**TABLE 2:** Multivariable logistic regression analysis showing the factors associated with undernutrition in slum-dwelling adults in Bangladesh^1^

Variables	OR	95% CI	*P* value	aOR	95% CI	*P* value
Age	0.96	0.93, 0.99	0.04	0.90	0.84, 0.96	0.001
Sex (female)	1.85	1.05, 3.31	0.03	0.61	0.20, 1.87	0.39
No. of siblings	1.27	1.10, 1.47	0.002	1.39	1.11, 1.77	0.005
Family income	0.99	0.99, 0.99	0.004	0.99	0.99, 1.00	0.30
Money spent on food	0.99	0.99, 0.99	0.01	0.99	0.99, 1.00	0.58
Employed (yes)	0.61	0.37, 1.01	0.06	1.03	0.41, 2. 60	0.95
Screen hours	1.23	1.05, 1.45	0.01	1.09	0.82, 1.45	0.55
SRQ-20 score	1.08	1.03, 1.14	0.01	1.12	1.04, 1.23	0.006
DDS	0.80	0.66, 0.97	0.02	0.75	0.56, 0.99	0.04
Always wash hands before meal	0.46	0.24, 0.86	0.02	0.33	0.12, 0.87	0.03
Always wash hands after toilet	1.54	0.89, 2.69	0.13	3.73	1.48, 9.92	0.006
Use of toilet paper	0.47	0.27, 0.82	0.01	0.52	0.22, 1.20	0.13
CRP (mg/L)	0.88	0.80, 0.94	0.001	0.82	0.73, 0.92	0.001
AGP (mg/dL)	0.99	0.98, 0.99	0.01	1.00	0.99, 1.02	0.79
MPO (ng/mL)	1.01	1.01, 1.01	0.03	1.00	0.99, 1.00	0.13
AAT (mg/g)	7.50	2.54, 24.64	0.001	4.82	1.01, 25.29	0.049
*H. pylori* (yes)	0.15	0.09, 0.26	<0.001	0.11	0.05, 0.23	<0.001
Anemia (yes)	2.58	1.55, 4.33	<0.001	3.63	1.62, 8.58	0.002
Iron deficiency (yes)	1.79	0.89, 3.73	0.11	0.77	0.26, 2.21	0.62
Zinc deficiency (yes)	1.55	0.94, 2.57	0.09	1.98	0.90, 4.49	0.10

1 Multivariable logistic regression model was adopted and adjusted for the variables with *P* values <0.20 in the univariate logistic regression analysis. AAT, α-1 antitrypsin; AGP, α-1-acid glycoprotein; aOR, adjusted odds ratio; CRP, C-reactive protein; DDS, dietary diversity score; MPO, myeloperoxidase; SRQ-20, self-reporting questionnaire-20.

## Discussion

Using a case-control study design to identify the factors associated with undernutrition in adults living in an urban slum in Mirpur, Bangladesh, we found that the number of siblings, SRQ-20 score, always washing hands after using the toilet, increased fecal concentrations of AAT, and anemia were significantly positively associated with adult undernutrition. In contrast, age, DDS, always washing hands before cooking or taking a meal, increased plasma CRP concentrations, and *H. pylori* infection were associated with reduced risk of low BMI. However, some of the factors identified in other studies, for example, sex, education, employment status, family income, and smoking (24, 27, 41, 42), were not associated with undernutrition in the adults enrolled in this study.

The past 3 BDHS reports showed that the prevalence of adult undernutrition in Bangladesh is higher in younger women (23, 43, 44). A study conducted in Uganda found that younger male study participants were more likely to be underweight (45). Similarly, a nationwide survey conducted in Iran reported that undernourished adults were more likely to be younger than normal-weight adults (46). Consistent with these findings, our results revealed an inverse relationship between age and having a BMI <18.5. We also observed that the odds of undernutrition were higher among adults who had a higher number of siblings. This finding could be due to the fact that an increase in the number of siblings can lead to lower allocation of foods and other resources to the individuals within a family, particularly in countries similar to Bangladesh, where cohabitation of extended families is more prevalent.

Dietary diversity, linked with micronutrient density of the diet, is indicative of food security and has been found to have significant association with improved nutrient intake (47). Prior reports confirmed that low dietary diversity is associated with undernutrition, both in adolescents and adults (48, 49). The inverse relationship between DDS and undernutrition among the enrolled participants supports the findings of previous studies conducted in different parts of the world (48, 50). Evidence suggests that micronutrient deficiency is associated with malnutrition in both adults and children (51, 52). Anemia has been associated with poor appetite and low BMI (53, 54). In our study, the prevalence of anemia was more pronounced among the undernourished adults than in the control group. Moreover, the anemic participants were nearly 4 times more likely to have undernutrition than participants with a normal BMI (18.5–24.9). Our results are in agreement with prior reports, as well as with findings from national surveys showing that underweight women had a higher likelihood of being anemic (55).

The current analysis revealed that the likelihood of being undernourished was higher among patients who had a higher SRQ-20 score. Previously, childhood abuse and eating disorders were found to be associated with nutritional status during adulthood (56). Studies conducted in the Netherlands and rural Bangladesh showed an association between depressive symptoms and undernutrition among older adults (57, 58). Depression is considered to be the prodromal stage of dementia and has also been proposed to be a risk factor of developing dementia (59). Study findings revealed that underweight adults were more likely to have dementia, which may persist for as long as 15 y after underweight status is recorded (60). Both dementia and depression were found to be linked with weight loss as well as poor nutrition in nursing home patients (61). Moreover, depression has been reported to be a significant determinant of low BMI in community-dwelling adults (62–64). In accordance with those findings, our results also corroborate the association between depressive symptoms as measured with the SRQ-20 score and undernutrition among the slum-dwelling adults.

Poor hygiene practice is an important factor contributing to altered immune function, increased risk of infection, and undernutrition (65, 66). Hand washing reduces transmission of pathogens and is associated with a reduction in undernutrition (67, 68). In the current study, the odds of being undernourished were significantly lower in adults who always washed their hands before cooking or eating than in those who did not. Surprisingly, we observed that the practice of always washing hands after using the toilet was positively associated with undernutrition in our cohort. This finding is counterintuitive and underscores the necessity of further exploration. The prevalence of *H. pylori* infection was higher among the healthy participants enrolled in this study. There is conflicting evidence on the relationship between *H. pylori* infection and nutritional status. *H. pylori* is a cause of dyspepsia and gastritis, which can lead to anorexia and weight loss (69, 70). Moreover, *H. pylori* infection was found to be correlated negatively with adult BMI in a study conducted on hospitalized patients (71). In contrast, a number of studies have demonstrated a positive association between *H. pylori* infection and BMI in adults (72–74).

We observed that the odds of undernutrition were lower among those with higher plasma concentrations of CRP. This finding is in line with previous reports showing lower CRP concentrations in underweight US adults (75, 76). CRP is an acute-phase reactant and known to have positive correlation with adult BMI (77, 78). Data from a recent study showed that reduction in CRP is associated with weight loss in adult women (79). Experiments in animal models exhibited that undernutrition can attenuate the acute inflammatory response and leads to hyporesponsiveness of acute-phase reactants, including CRP (80, 81). However, the interaction between undernutrition and systemic inflammation is a complex phenomenon and the causal relationships as well as the biological mechanisms are not well understood, with genetics, age, diet, and lifestyle all likely to play a role (82). In all of the study participants, concentrations of fecal MPO and AAT, biomarkers of altered gut health, were much higher than those reported in subjects in nontropical settings (40). This finding is suggestive of the widespread presence of chronic gastrointestinal inflammation and altered intestinal permeability in our adult cohort. However, AAT concentrations were significantly elevated in the undernourished adults compared the control group, with almost 5 times increased odds of being undernourished among adults with elevated AAT concentration in the stools compared with the healthy adults. Elevated AAT concentration in stool is a potential marker of increased intestinal permeability and indicates enteric protein loss (83). It is evident that increased intestinal protein loss contributes to malnutrition in both children and adults (84). A multicountry birth cohort study conducted across 3 continents revealed a positive association between increased fecal concentration of AAT and childhood undernutrition (85).

A limitation of this study includes the single-point collection of data, which reduced our ability to capture potentially dynamic relationships between factors associated with undernutrition in adults. Moreover, reporting and recall biases are also potential limitations of the study. We additionally lacked information on exposure to intestinal pathogens, presence of gastrointestinal symptoms, and morbidity status of the study participants, information that would have allowed us to determine whether these variables were implicated in undernutrition among slum-dwelling adults. Lastly, the sample size was relatively small for detecting the relevant associations between explanatory variables and outcomes. Our study also has several strengths. To our knowledge, this is the first study to investigate the association of diverse factors, including dietary diversity, micronutrient deficiencies, psychological symptoms, and biomarkers of systemic inflammation, as well as altered gut health, with undernutrition among slum-dwelling adults in Bangladesh. Furthermore, the statistical modeling and postestimation diagnostic methods used for this analysis were appropriate and robust. The 89% predictive power of the ROC curve signifies that the accuracy of the model applied in this analysis was excellent. It also indicates that variables included in this model perfectly correspond to the correlates of adult undernutrition for this slum-dwelling population.

In conclusion, the results of this case-control study suggest that undernutrition in slum-dwelling adults in Mirpur, Bangladesh, has a complex etiology, with correlates found in several domains. Our findings revealed that the number of siblings, SRQ-20 score, always washing hands after using the toilet, higher fecal concentration of AAT, and anemia had significant positive associations with adult undernutrition, while age, DDS, always washing hands before cooking or taking meals, increased CRP concentrations, and *H. pylori* infection were negatively associated with BMI. Our data are also consistent with the existence of subclinical gut enteropathy among adults living in slums in Bangladesh. Future interventional studies should investigate the causal role of the factors identified in this study to determine their relative contributions to undernutrition in slum-dwelling adults.

## Supplementary Material

nqz327_Supplemental_Figures

## Notes

Supported by the Bill and Melinda Gates Foundation under its Global Health Program, Project investment ID OPP1136751. (https://www.gatesfoundation.org/How-We-Work/Quick-Links/GrantsDatabase/Grants/2015/11/OPP1136751). The funders had no role in the study design; collection, analysis, and interpretation of data; preparation, review, or approval of the manuscript; and decision to submit and publication of the manuscript.

Supplemental Figure 1–3 are available from the “Supplementary data” link in the online posting of the article and from the same link in the online table of contents at https://academic.oup.com/ajcn/.

Abbreviations used: AAT, α-1 antitrypsin; AGP, α-1-acid glycoprotein; aOR, adjusted odds ratio; BDHS, Bangladesh Demographic and Health Survey; CED, chronic energy deficiency; CRP, C-reactive protein; DDS, dietary diversity score; MPO, myeloperoxidase; NEO, neopterin; SRQ-20, self-reporting questionnaire-20; ROC, receiver operating characteristic; VIF, variance inflation factor.
