# Supplementary material for: Evidence of gut enteropathy and factors associated with undernutrition among slum-dwelling adults in Bangladesh
Source: Am J Clin Nutr. 2020 Jan 7;111(3):657–66. doi: 10.1093/ajcn/nqz327 (PMC7049527; doi:10.1093/ajcn/nqz327)

**Supplemental Figure 1.** Proportions of participants with elevated level of fecal biomarkers among slum-dwelling adults enrolled in this study. To determine the elevated levels of fecal biomarkers, we used the cut-offs set for non-tropical countries (<2,000 ng/ml, <70 nmol/l, <0.27 mg/g, respectively for MPO, NEO, and AAT). The differences for AAT and NEO showed statistical significance (p-values are 0.001 and 0.02, respectively). Abbreviations used: AAT, alpha-1 antitrypsin; MPO, myeloperoxidase; NEO, neopterin.

**p = 0.001**

**p = 0.09**

**p = 0.02**

**Supplemental Figure 2.** Multi-panel scatter plots showing the correlations of different biomarkers with body mass index (BMI) of the adult participants both in healthy and undernourished groups. The dotted red lines indicate the cut-off set for BMI to differentiate between healthy and undernourished adults as well as the reference values of the respective biomarkers. ‘*rho*’ represents the spearman’s correlation coefficient between BMI and the individual biomarker, and “p” indicates the statistical significance. Abbreviations used: AAT, alpha-1 antitrypsin; AGP, Alpha-1-acid glycoprotein; CRP, C-reactive protein; MPO, myeloperoxidase; NEO, neopterin. The cut-offs used in the figure: BMI, 18.5 kg/m^2^; Hemoglobin, 12 g/dl; Ferritin, 12 ng/ml; Zinc, 0.7 mg/L MPO, 2,000 ng/ml; NEO, 70 nmol/l; AAT, 0.27 mg/g; CRP, 5 mg/l; AGP, 100 mg/dl.

**
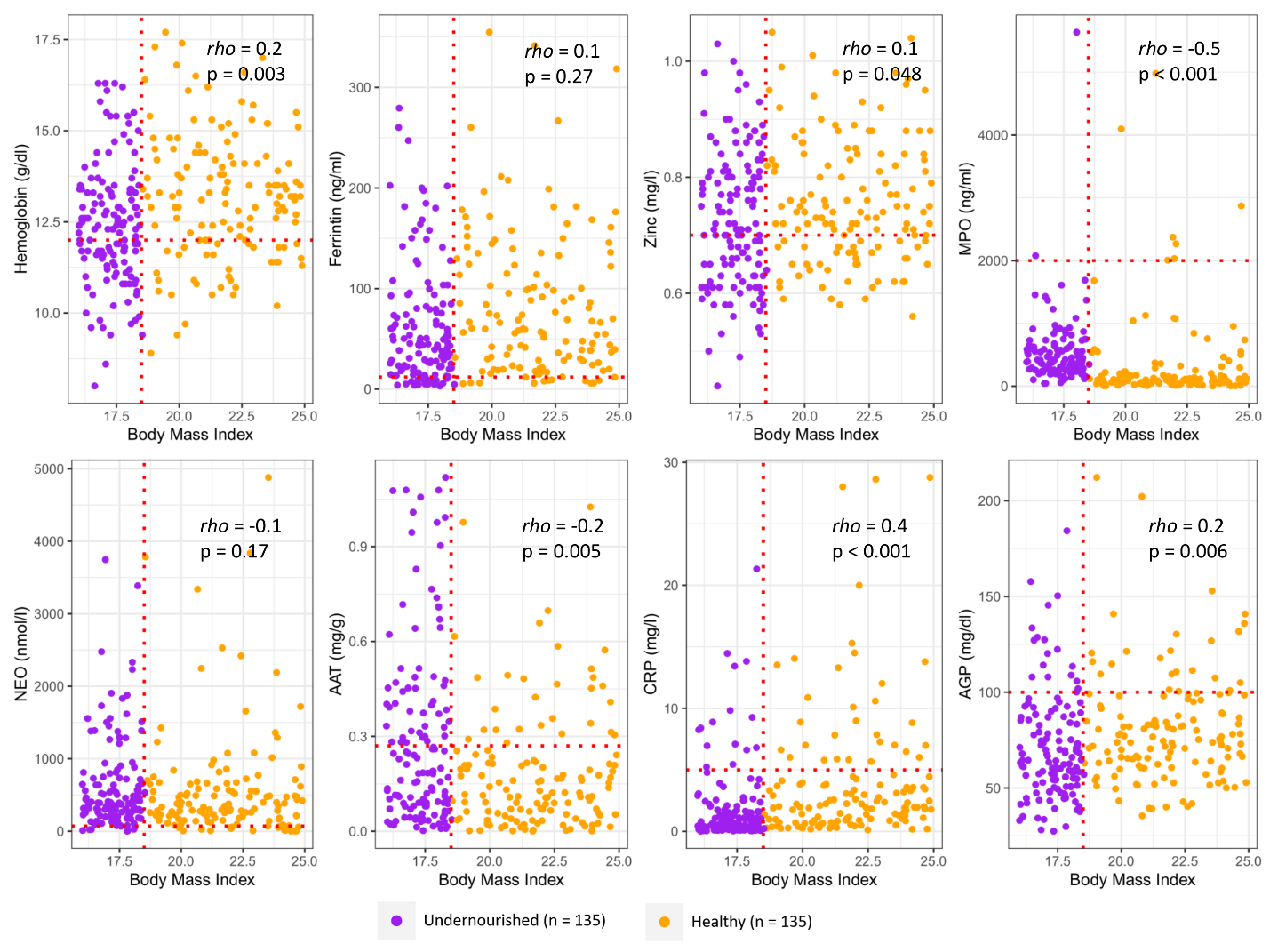
**

**Supplemental Figure 3.** Receiver operating characteristic (ROC) curve of fitted logistic regression model to measure the accuracy of the model. The ROC curve illustrated that the overall predictive power of the estimated logistic regression model was 89.2%.


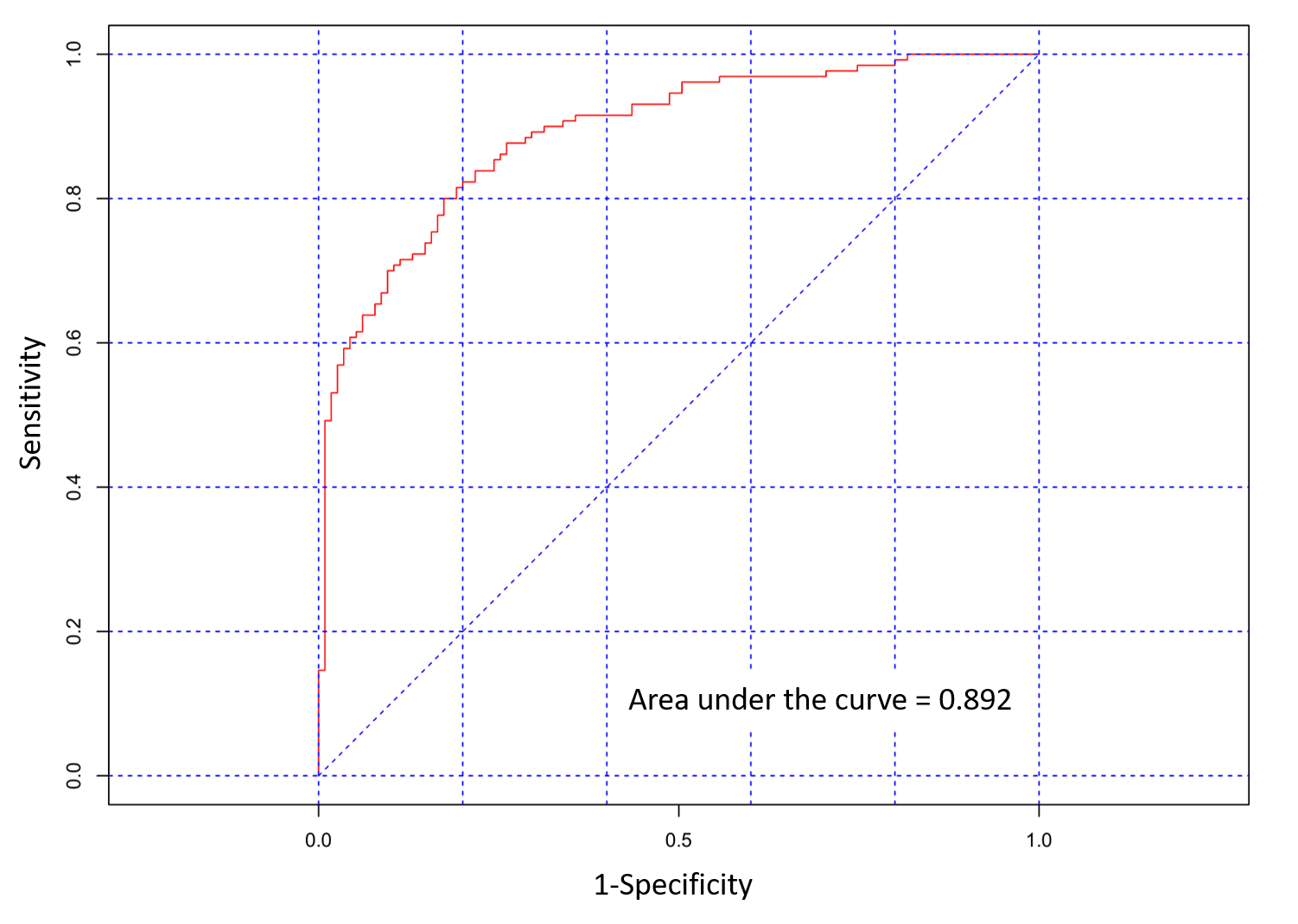

Supplement: nqz327_Supplemental_Figures [file nqz327_supplemental_figures.docx]
